# Theoretical and Experimental Study of the Photocatalytic Properties of ZnO Semiconductor Nanoparticles Synthesized by *Prosopis laevigata*

**DOI:** 10.3390/ma16186169

**Published:** 2023-09-12

**Authors:** Mizael Luque Morales, Priscy Alfredo Luque Morales, Manuel de Jesús Chinchillas Chinchillas, Víctor Manuel Orozco Carmona, Claudia Mariana Gómez Gutiérrez, Alfredo Rafael Vilchis Nestor, Rubén César Villarreal Sánchez

**Affiliations:** 1 Facultad de Ingeniería Arquitectura y Diseño, Universidad Autónoma de Baja California, Ensenada 22860, Mexico; mizael.luque@uabc.edu.mx (M.L.M.); pluque@uabc.edu.mx (P.A.L.M.); cmgomezg@uabc.edu.mx (C.M.G.G.); 2 Departamento de Ingeniería y Tecnología, Universidad Autónoma de Occidente, Guasave 81048, Mexico; manuel.chinchillas@uadeo.mx; 3 Departamento de Metalurgia e Integridad Estructural, Centro de Investigación en Materiales Avanzados, Chihuahua 31136, Mexico; 4 Centro Conjunto de Investigación en Química Sustentable, UAEM-UNAM, Toluca 50200, Mexico; arvilchisn@uaemex.mx

**Keywords:** zinc oxide, Langmuir–Hinshelwood–Hougen–Watson model, photocatalysis, methylene blue

## Abstract

In this work, the photocatalytic activity of nanoparticles (NPs) of zinc oxide synthetized by *Prosopis laevigata* as a stabilizing agent was evaluated in the degradation of methylene blue (MB) dye under UV radiation. The theoretical study of the photocatalytic degradation process was carried out by a Langmuir–Hinshelwood–Hougen–Watson (LHHW) model. Zinc oxide nanoparticles were synthesized by varying the concentration of natural extract of Prosopis laevigata from 1, 2, and 4% (weight/volume), identifying the samples as ZnO_PL1%, ZnO_PL2%, and ZnO_PL4%, respectively. The characterization of the nanoparticles was carried out by Fourier transform infrared spectroscopy (FT-IR), where the absorption band for the Zn-O vibration at 400 cm^−1^ was presented; by ultraviolet–visible spectroscopy (UV–vis) the value of the band gap was calculated, resulting in 2.80, 2.74 and 2.63 eV for the samples ZnO_PL1%, ZnO_PL2%, and ZnO_PL4%, respectively; XRD analysis indicated that the nanoparticles have a hexagonal zincite crystal structure with an average crystal size of 55, 50, and 49 in the sample ZnO_PL1%, ZnO_PL2%, and ZnO_PL4%, respectively. The morphology observed by TEM showed that the nanoparticles had a hemispherical shape, and the ZnO_PL4% sample presented sizes ranging between 29 and 45 nm. The photocatalytic study showed a total degradation of the MB in 150, 120, and 60 min for the samples ZnO_PL1%, ZnO_PL2%, and ZnO_PL4%, respectively. Also, the model explains the experimental observation of the first-order kinetic model in the limit of low concentrations of dye, indicating the influence of the mass transfer processes.

## 1. Introduction

Water is essential for the survival of all living organisms. There is a wide range of harmful organic contaminants present in water organisms, such as pesticides, phenols, detergents, pharmaceuticals, carbohydrates, and organic dyes, among others. In general, water pollution causes eutrophication of plants, damage to aquatic systems, and severe threats to human health, causing diseases [1,2,3]. Of all the pollutants mentioned above, dyes are an important class of pollutants in the effluents of various industries, such as textile, plastics, food, and paper [4]. The presence of dyes in water, even in minute quantities, is undesirable since most are toxic, mutagenic, and carcinogenic [5].

There are many methods for removing organic pollutants from wastewater, each with its characteristics [6]. However, photocatalytic degradation has been reported to be an environmentally friendly option with much potential. In recent years, photocatalysis has gained significant interest in treating waters with industrial pollutants due to its advantages over other methods, such as waste disposal, low cost, and low environmental impact, among others [7,8,9]. Among the materials that have been used as photocatalysts, some of the most common are semiconductors, as they have band gap values ranging from 1.5 eV to 3.5 eV, allowing them to conduct electrons in the presence of light [10].

Zinc oxide (ZnO) is a semiconductor material that has attracted significant attention and has become well known for its various advantages over other semiconductor nanoparticle oxides, including low toxicity, ease of availability, chemical stability, and low cost [11,12,13]. Among the applications that stand out for zinc oxide are: anticancer, antibacterial, and antidiabetic activities, photocatalysis, cosmetics, solar energy harvesting, sensors, pigments, optical filters, sunscreens, dye-sensitized solar cells, etc. [14,15,16,17,18]. Currently, there are various routes for the synthesis of ZnO nanoparticles, such as physical methods (mechanical milling, laser ablation, sputtering, among others) and chemical methods (pyrolysis, sol gel, photochemical reduction, among others) [19,20]. However, most of these methods are expensive, require sophisticated equipment, require high energy consumption, use reagents that are harmful to the environment, generate secondary waste, etc. Another method that has been used in recent years is green synthesis. This process is cheap, fast, and uses natural materials such as leaves, fruit shells, flowers, or stems of plants and trees to reduce metallic salts and obtain nanoparticles with desired characteristics [21]. Using plant extracts brings advantages such as high availability, safety, non-toxic (in most cases), and various phytochemicals that help reduce metal salts and provide stability to nanoparticles [22].

The synthesis of this material by using green chemistry methods has gained importance in recent years. Kamarajan et al., 2022, reported the green synthesis of nanoparticles using *Acalypha indica* showing promising results in the degradation of methylene blue [23]. Sasi et al., 2022, worked on the synthesis of nanoparticles using *Garcinia cambogia* demonstrating promising results in photocatalytic activity degrading methylene blue, crystal violet and phenol red [24]. Finally Vu Nu et al., 2022, reported the use of *Cordia myxa* for the synthesis of nanoparticles and good results were obtained in the photocatalytic degradation of methylene blue [25], among other related works. The mesquite tree (*Prosopis laevigata*) is one of the primary natural resources of the United States and Mexico. It is widely used as animal feed, to obtain wood and coal, and it is used in the construction industry and medicine, among other applications. It has been reported that *Prosopis laevigata* contains sugars, proteins, carbohydrates, fats, crude fiber, phenolic compounds, and polyphenols, among others [26,27,28]. These phytochemicals actively contribute to the formation of nanoparticles through green synthesis.

What has not yet to be reported in the literature is the use of *Prosopis laevigata* extract in the synthesis of ZnO nanoparticles, nor the evaluation of a theoretical model on the photocatalytic behavior of these nanomaterials. Therefore, in this work, *Prosopis laevigata* extract has been used as a reducing agent in the green synthesis of ZnO nanoparticles, which proved their photocatalytic effectiveness in the degradation of methylene blue (MB) by ultraviolet radiation. Also, the theoretical modeling of the photocatalytic process by a Langmuir–Hinshelwood–Hougen–Watson (LHHW) approach was carried out, and variations in the dye concentration were performed to study the effect of mass transfer phenomena on the degradation kinetics.

## 2. Materials and Methods

### 2.1. Materials

For the green synthesis of ZnO NPs, *Prosopis laevigata* (mesquite) leaves were used as a reducing agent (purchased locally), zinc nitrate, Zn(NO_3_)_2_∙6H_2_O, as precursor salt (purchased from Faga Lab, Los Mochis, Mexico), and deionized water as solvent.

### 2.2. Methodology

#### 2.2.1. Preparation of Extract

For the extraction process of the reducing agent, *Prosopis laevigata* extracts were prepared at concentrations of 1, 2, and 4% (*w*/*v*) in 50 mL of deionized water. Previously, *P. laevigata* leaves were cleaned, crushed, and added in deionized water and kept under magnetic agitation for 2 h. Subsequently, they were placed in a thermal bath at 60 °C for 1 h. Finally, they were vacuum filtered using a Watman #4 filter. The extract was stored for later use.

#### 2.2.2. Synthesis of ZnO Nanoparticles

**Figure 1:**
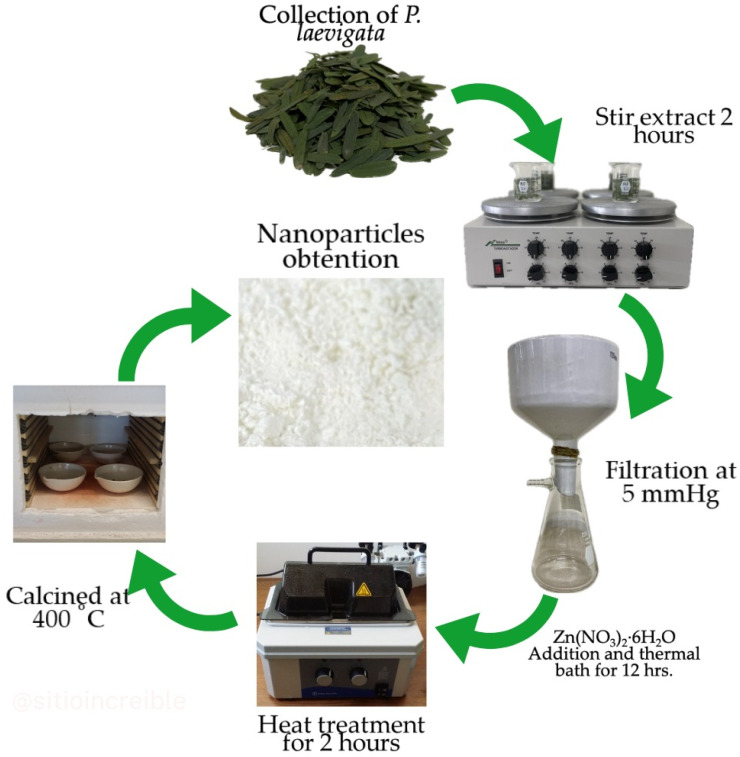
Green synthesis process of ZnO nanoparticles using *Prosopis laevigata* extract.

To synthesize ZnO NPs, 2 g of zinc nitrate was added to 42 mL of *P. laevigata* extract (amount of extract obtained after the filtration process). The solutions were magnetically stirred for 1 h at room temperature. Subsequently, the samples were placed in a thermal bath at 60 °C for 13 h until most of the water evaporated and a high viscosity consistency was obtained. Finally, the samples were calcined at 400 °C for 1 h. Figure 1 shows the process carried out in the green synthesis of ZnO nanoparticles using *Prosopis laevigata* extract.

#### 2.2.3. Characterization

Different techniques were used to study the physical, chemical, and optical properties of the synthesized ZnO NPs to characterize the material. In order to know the functional groups presents in the material, Fourier transform infrared spectroscopy (FT-IR) ATR mode was used, and the spectra were obtained with Perkin Elmer brand equipment (Waltham, MA, USA) at 0.5 cm^−1^ resolution and 400 to 3500 cm^−1^ measurement range. For the study of the optical properties and the band gap of the materials ultraviolet–visible (UV–vis) spectroscopy was used at a wavelength of 200 to 800 nm, using a Perkin Elmer UV/VIS Lambda 365 spectrometer. Crystal structure analysis was determined by X-ray diffractometry (XRD) using a Bruker D2-phase diffractometer (Billerica, MA, USA). The nanoparticles’ size, shape, and structure were determined using transmission electron microscopy (TEM) with a JEOL microscope (Tokyo, Japan).

#### 2.2.4. Photocatalytic Activity

The photocatalytic activity of ZnO NPs was analyzed to determine the degradation of methylene blue (MB) under ultraviolet (UV) radiation. The degradation process was carried out inside a stainless-steel reactor, with a length of 26.6 cm and a width of 5 cm, equipped with a UV lamp. To analyze the photocatalytic activity of the different samples, 50 mg of ZnO nanoparticles were added to 50 mL of the MB-contaminated solution (at 15 ppm); consecutively, the samples were shaken for 30 min in the dark to carry out the adsorption–desorption process. Subsequently, the suspensions were irradiated with UV light for 3 h. Aliquots were taken every 10 min during the first 30 min and every 30 min during the following 2 h 30 min. The percentage of MB degradation was determined by UV–vis spectroscopy.

#### 2.2.5. Model

The LHHW model for the degradation kinetic process is based on the following assumptions: (1) The number of adsorption sites on the surface is limited. (2) Each site can adsorb only one molecule, and a maximum of one layer can cover the catalyst surface. (3) The adsorption reaction can be reversible. (4) The catalyst surface is homogeneous. (5) There is no interaction between the adsorbed molecules [29,30,31,32]. For this purpose, two stages are taken into account during the degradation of the chemical species, the adsorption stage and the reaction stage, which are defined below:

Adsorption stage:

(1) $$A+l\begin{array}{c} K_{A}\\ ⇄\\ K_{d} \end{array}A.l$$

where A represents a dye molecule, *l* represents an active site on the catalyst surface, K_A_ and K_d_ are kinetic constants.

Reaction stage:

(2) $$A.l\begin{array}{c} K_{sr}\\ ⇄\\ K_{sr}^{\prime } \end{array}R.l$$

where A.*l* represents a molecule A adsorbed on a site *l*, and R.*l* represents the degraded product adsorbed on the catalyst, with K_sr_ and K′_sr_ as kinetic constants of the reaction.

The model states that the degradation rate (−*r_A_*) for the adsorbed chemical species A is [33]:

(3) $$-r_{A}=\frac{B\;C_{A}}{1+AC_{A}}$$

If the desorption process is compared to the adsorption process, then A = K_A_/K_sr_ and B = K_A_C_t_; C_A_ is the concentration of the dye around the catalyst; K_A_ the adsorption constant; and C_t_ the concentration of active sites on the catalyst surface.

Integrating Equation (1) and defining the variable X = C/C_A0_, where C_A0_ is the initial concentration, we have for the degradation time:

(4) $$t=-\frac{1}{B}\;\ln\left(\frac{1}{X}\right)+\frac{AC_{A0}}{B}\;\left(1-X\right)$$

For the calculation of the constants A and B, an objective function (3) is defined to minimize the difference between the value of X calculated by the model and the value of X measured experimentally.

(5) $$Fobj=\sum_{i=0}^{n}{\left(X_{calculated}-X_{measured}\right)}^{2}$$

where *n* is the number of experimental data and X_calculated_ is obtained from numerical solve of Equation (2). The minimization of the objective function is performed by means of the conjugate gradient method [34].

## 3. Results and Discussion

### 3.1. FTIR

**Figure 2:**
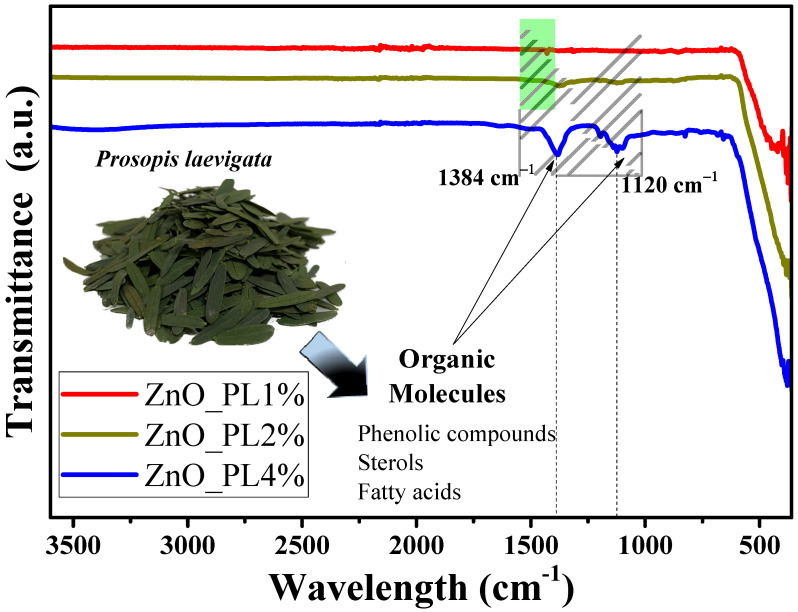
Study of functional groups with FT-IR of ZnO nanoparticles synthesized with different percentages (1, 2, and 4%) of *Prosopis laevigata*.

Figure 2 shows the FTIR spectra, analyzed in the range from 4000 cm^−1^ to 400 cm^−1^, for the ZnO_PL1%, ZnO_PL2%, and ZnO_PL4% samples. The materials show vibrations at 3410, 1385, 1120, and 400 cm^−1^. The band at 3410 cm^−1^ represents the vibration of the hydroxyl group (O-H) [35]. The bands at 1384 and 1120 cm^−1^ are attributed to the C-H and C-O vibrations of the carboxylic groups present in the organic molecules of the *Prosopis laevigata* extract [36,37]. Finally, the band observed at 400 cm^−1^ in the three samples analyzed is attributed to the stretching of metal–oxygen (Zn-O), which confirms the obtaining of ZnO nanoparticles [38].

### 3.2. Optical Properties

**Figure 3:**
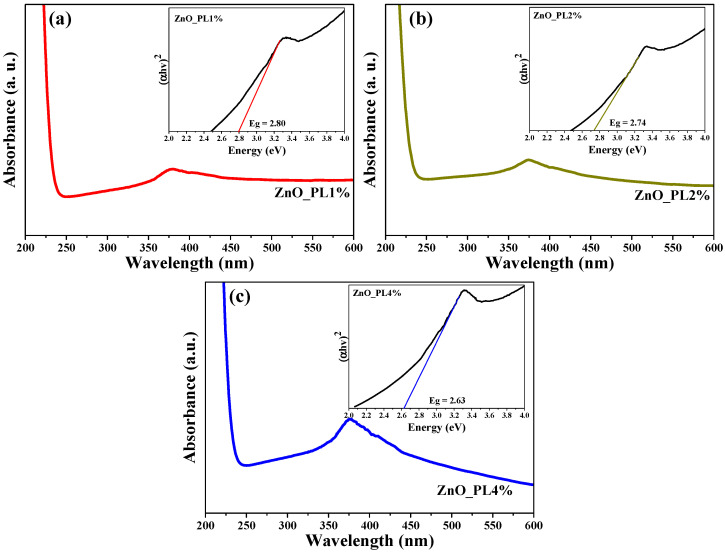
Study of optical properties and band gap ZnO. (**a**) ZnO_PL1%. (**b**) ZnO_PL2% and (**c**) ZnO_PL4%.

The determination of the optical properties of the material was carried out using UV–vis spectroscopy. Figure 3 shows the spectrum obtained for ZnO NPs dispersed in water, in a wavelength range from 200 nm to 600 nm, with a maximum absorption peak located around 350 nm for the 3 samples. The Tauc model was used to calculate the band gap.

(6) $${\left(\alpha \;h\nu \right)}^{1/n}=B\left(h\nu -Eg\right)$$

where α(ν) is the absorption coefficient (Lambert-Beer), hν is the photon energy, B is a proportionality constant (it is determined by the refractive index, the effective electron, and hole masses), Eg is the energy of the band gap and the value of *n* corresponds directly to the band gap semiconductor (*n* = 1/2) [39,40]. The calculated values of the band gap by the method of Tauc resulted in 2.80, 2.74, and 2.63 eV for ZnO_PL1%, ZnO_PL 2%, and ZnO_PL 4% of ZnO NPs, respectively. These values are very similar to those reported in the literature for ZnO NPs [41]. It should be noted that as the percentage of extract used for material biosynthesis increases, the band gap decreases. This decrease in the energy band gap is due to the size of the NPs; the greater the amount of extract used in biosynthesis, the more organic molecules remain on the surface of the nanoparticles (residual carbon) acting as photosensitizers [35,42,43].

### 3.3. XRD

**Figure 4:**
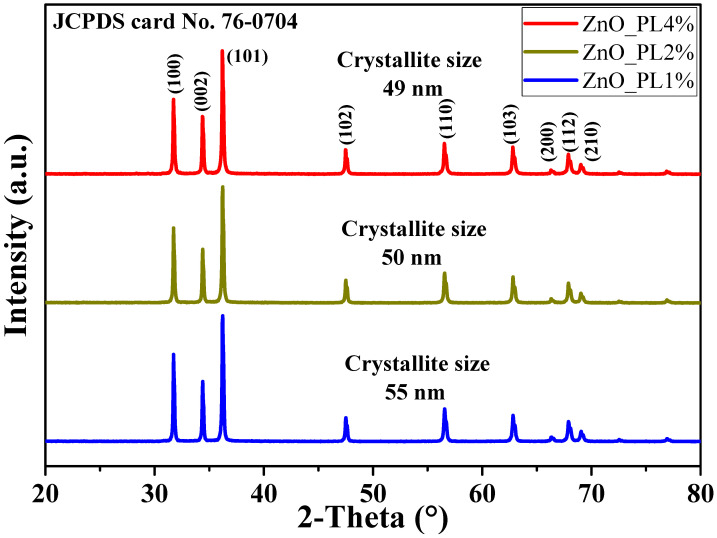
Study of the crystal structure of ZnO NPs.

Figure 4 shows the XRD analysis of the ZnO NPs. The characteristic peaks of this material are observed in the diffraction pattern, being identified at 31.73, 34.40, 36.23, 47.51, 56.56, 62.82, 66.42, 67.92°, and 69.04 2θ, corresponding to Miller indices of (100), (002), (101), (102), (110), (103), (200), (112), and (210), respectively. These peaks coincide with those of the JCPDS Card No. 76-0704, which describes the ZnO NPs as hexagonal structures type zincite [35]. To calculate the crystallite size, the Debye–Scherrer formula (5) was used for the three most intense peaks (100), (002), and (101):

(7) $$L=\frac{0.9\;\lambda }{\beta \cos\theta }$$

where *L* is the crystallite size (nm), λ the incident wavelength, β the full width at half maximum of the peak, θ the Bragg angle [44]. The results showed that the size of the crystallites was 55, 50, and 49 nm for samples ZnO_PL1%, ZnO_PL2%, and ZnO_PL4%, respectively. The size obtained in this study is within the range of previous reports in the literature for ZnO nanoparticles [38]. Research has demonstrated that using more extract in green synthesis results in smaller crystallites. This is due to the organic molecules acting as a barrier and preventing the nanomaterial from agglomerating and growing, according to a study [30].

### 3.4. TEM

**Figure 5:**
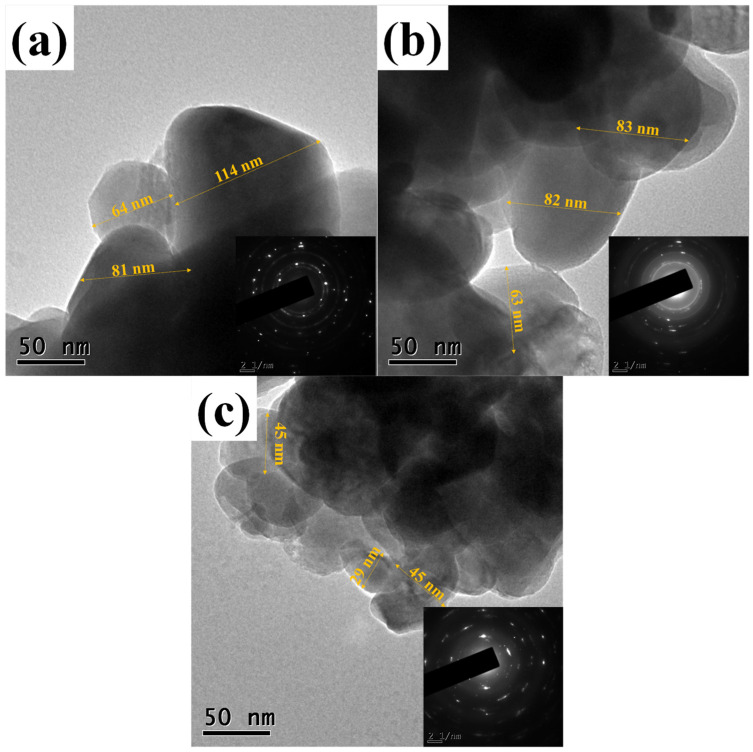
Morphology of the nanoparticles by TEM, (**a**) ZnO_PL1%, (**b**) ZnO_PL2%, and (**c**) ZnO_PL4%.

Figure 5 shows the morphology of ZnO NPs by TEM. TEM micrographs (a), (b), and (c) show a semi-spherical shape of different sizes for the three ZnO samples, with small agglomerations. The imageJ (version 1.6.0) software was used to measure nanoparticles observed in the micrographs. The ZnO_PL1% NPs showed sizes between 64 and 114 nm. The ZnO_PL2% sample presented particle sizes between 63 and 83 nm, and finally, ZnO_PL4% presented measurements between 29 and 45 nm. The effect caused by the concentration of *P. laevigata* extract during the synthesis of ZnO on the nanoparticle size is evident; it is observed that the higher the concentration of extract the nanoparticle size is lower, as previously reported in the literature [45,46]. Furthermore, the crystalline nature of the ZnO nanoparticles was confirmed with the selected area electron diffraction (SAED) pattern. The ring-shaped diffraction pattern demonstrates that the nanoparticles are nanocrystalline [47,48].

### 3.5. Formation Mechanism of ZnO Nanoparticles

**Figure 6:**
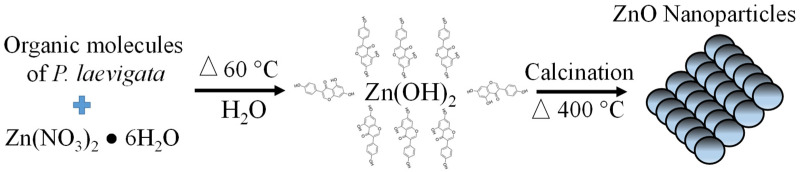
Proposal for a mechanism for the formation of biosynthesized ZnO nanoparticles with *P. laeveigata*.

Figure 6 presents a proposed mechanism for forming ZnO nanoparticles biosynthesized with *P. laevigata* extract. The nanoparticle formation reaction begins with the hydrolysis of the metal precursor (zinc nitrate) when in contact with the *P. laevigata* extract. Subsequently, the reaction continues with nucleation and later agglomeration, leading to the formation of ZnO nanoparticles [49]. In the formation of the nanomaterial, the organic molecules present in the extract are functionalized on the surface of the nanoparticles with interactions with the OH groups of the extract [50]. These functionalized molecules help as stabilizing agents and prevent excessive growth of the nanoparticles [51].

### 3.6. Model

**Figure 7:**
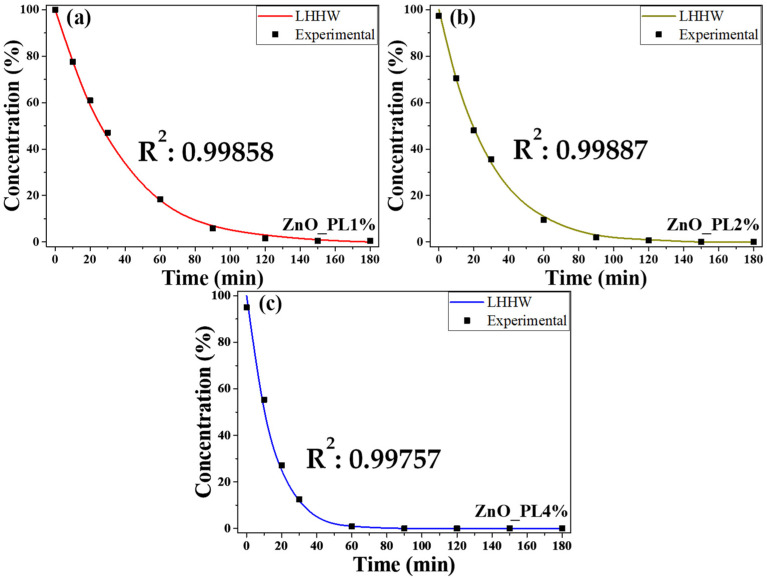
Comparison of degradation capacity of nanoparticles from different extract concentrations in the synthesis, (**a**) ZnO_PL1%, (**b**) ZnO_PL2%, and (**c**) ZnO_PL4%.

Figure 7 shows degradation graphs, where a nice fitting of the LHHW model with the experimental data can be observed. According to the model, the value of the constant B is related to the concentration of active sites on the surface of the NPs. The ZnO_PL1% sample, which has the largest size of NPs, has a value of the constant equal to 0.0328, while the samples of ZnO_PL2% and ZnO_PL4% have a value of B of 0.0527 and 0.0757, respectively. This increase in the value of constant B highlights an increase in the number of active sites when the size of the NPs decreases, which increases their reactivity, as can be seen with the decrease in degradation time. Figure 7a shows that the time to degrade 90% of the dye is 70 min for the sample of ZnO_PL1%, while in Figure 7b a time of 45 min is obtained for the sample of 2%. Finally, Figure 7c shows a time of 31 min for the sample of ZnO_PL4%. This increase in reactivity is also proven with the value of constant A, which according to the model is related to the adsorption and reaction constants. By decreasing the value of the constant A, from a value of 0.0216 for the sample of ZnO_PL1% to a value of 0.01 for the samples of ZnO_PL2% and ZnO_PL4%, we observe an increase in the kinetic constant of the reaction carried out on the surface of the catalyst, as the size of the NPs decreases. On the other hand, the speed of the photocatalytic reaction of the nanoparticles was 0.0305, 0.0387, and 0.0771 for the ZnO_PL1%, ZnO_PL2%, and ZnO_PL4% samples, respectively. Similar values have been found in the literature, for example, the work of Ludmila Motelica et al. in 2022 where they synthesized ZnO nanoparticles using various alcohols, reporting reaction rates of the nanoparticles between 0.0569 and 0.0935 [52]. In the present study, the ZnO_PL4% sample was the one that reached a higher reaction rate, which is attributed to the percentage of extract used in the biosynthesis. Some authors mention that the sharp edges in the nanoparticles lead to an improvement in the photocatalytic activity. In addition, the smaller the size of the nanoparticles, the greater the number of defects on the nanoparticle surface because there is a greater surface area. These defects represent catalytic centers for the photodegradation of polluting molecules, leading to improved photocatalytic activity [40,52,53].

**Figure 8:**
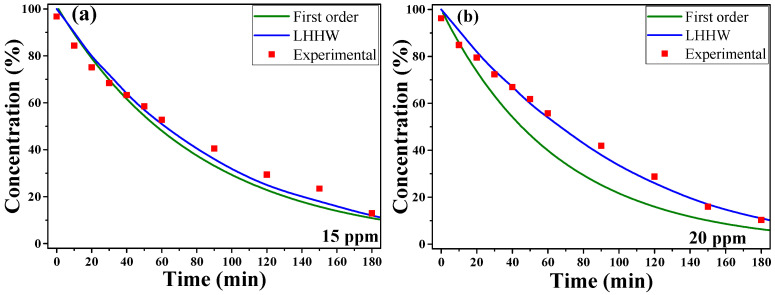
Comparison of degradation capacity of nanoparticles from variation in dye concentration, (**a**) 15 ppm and (**b**) 20 ppm.

In order to study the effect of mass transfer resistance of the dye to the catalytic surface, a variation in the amount of MB was made with the largest nanoparticles (1%), which is important for the control of the pressure drop in the design of packed bed reactors. Figure 8a shows the degradation curve for 15 ppm of dye concentration. It can be seen how the LHHW model fits the experimental data, but so does the first-order kinetic model *(−r_A_* = *BC_A_*), since at low dye concentrations (at the limit when it tends to zero), the LHHW model tends to the first-order kinetic model, explaining the observed convergence and the apparent kinetic order shown. The degradation curve for 20 ppm of dye is observed in Figure 8b. The graph shows how the LHHW model adjusts to the experimental data, but in these conditions of dye concentration, a kinetic of the first order is no longer observed, due to the increase in dye concentration in the solution [54]. The adjustment of the constant A under these conditions generates an A value of 0.01 for the sample of 15 ppm and a value of 0.0276 for the sample of 20 ppm. The increase in the value of constant A shows an increase in the kinetic constant of adsorption, due to the mass transfer resistance of dye molecules towards the catalytic surface.

### 3.7. Reaction Mechanism

**Figure 9:**
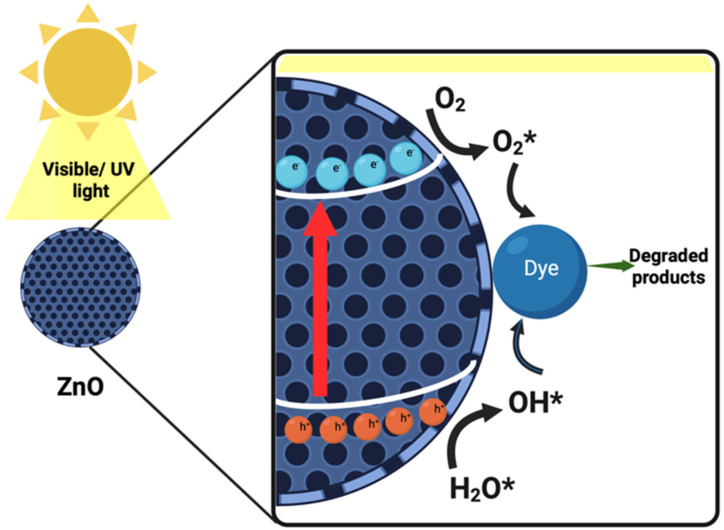
Reaction mechanism of nanoparticles in pollutant degradation.

Free radicals are essential for degrading dyes due to their high oxidizing power that can destroy harmful organic contaminants [55]. The reaction mechanism for the photocatalytic reaction is shown below in Figure 9.

The photocatalysis reaction of the organic dyes happens in a homogenous way; this implies that the dye molecules remain adsorbed on the catalyst surface (active sites). When UV light is irradiated over the system, electrons (e^−^) are generated in the conduction band and holes (h^+^) in the valence band [56]. The reactions in the surface are described as follows [55]:

Photon absorption

(8) $$ZnO+hv\left(UV\right)\to h_{VB}^{+}+e_{CB}^{-}\;\left(ZnO\right)$$

Reduction of O_2_ to superoxide radicals $O_{2}^{-}$

(9) $$O_{2}+e_{CB}^{-}\to O_{2}^{-}{}^{*}$$

Oxidation of hydroxyl ions and water molecules to generate hydroxyl radicals

(10) $$h_{VB}^{+}+OH^{-}\to OH^{*}$$

The holes (h^+^) react with water and, upon oxidation, generate hydroxyl radicals

(11) $$h_{VB}^{+}+H_{2}O\to OH^{*}+H^{+}$$

Radicals OH* and $O_{2}^{-}$* are responsible for the photodegradation of dyes to degraded products.

(12) $$Dye+OH^{*}\to \mathrm{intermediates}\;\to \;\mathrm{degraded}\;\mathrm{products}$$

(13) $$Dye+O_{2}^{-}{}^{*}\to \mathrm{intermediates}\;\to \;\mathrm{degraded}\;\mathrm{products}$$

## 4. Conclusions

In this work, we report the green synthesis of ZnO NPs using *Prosopis laevigata* extract with a simple and environmentally friendly process, as well as the photocatalytic degradation of methylene blue on the NPs. In addition, the degradation kinetics are described by the LHHW model, in order to study the photocatalytic and mass transfer processes. The LHHW model nicely fits the experimental data, evidencing the stages assumed in the photocatalytic process. The size of the NPs was varied using different concentrations of *P. laevigata* as a reducing and stabilizing agent. The LHHW model demonstrates that by decreasing the nanoparticle size, the number of active sites increases, generating greater reactivity. In the limit of low concentrations, first-order kinetics and the LHHW model coincide, showing that the resistance to the mass transfer of molecules generates an apparent degradation kinetic order of one. This research shows that it is possible to use an extract of *P. laevigata* as a reducing and stabilizing agent in forming ZnO nanoparticles. In addition, this study presents a theoretical model that adapts to a high percentage of the experimental. This work can help develop industrial catalytic reactors that help reduce pollution in bodies of water around the world.

## Data Availability

Data sharing is not applicable to this article.
